# Aneurismas da artéria e da veia braquial induzidos por uso contínuo de muleta: relato de caso

**DOI:** 10.1590/1677-5449.005317

**Published:** 2017

**Authors:** Débora Louise Lopes da Costa, Geraldo Felipe, Marcos Aurélio Perciano Borges

**Affiliations:** 1 Hospital de Base do Distrito Federal – HBDF, Unidade de Cirurgia Vascular e Angiologia, Brasília, DF, Brasil.

**Keywords:** lesões do sistema vascular, aneurisma, artéria braquial

## Abstract

O aneurisma arterial induzido por uso de muleta é um evento raro, e a associação com aneurismas venosos não está descrita na literatura. Relatamos o caso de uma paciente que, após o uso prolongado dessa órtese, apresentou quadro de isquemia aguda de membro superior secundária à trombose de um aneurisma da artéria braquial, associado ao achado incidental de aneurismas da veia braquial. Embora a principal causa de oclusão arterial aguda de membro superior seja a embolização de fonte cardíaca, deve-se considerar a possibilidade de embolização arterioarterial por aneurismas provocados pelo uso prolongado de muletas. Os aneurismas venosos também devem ser suspeitados, uma vez que podem ser sede de trombos e fonte de êmbolos pulmonares.

## INTRODUÇÃO

O uso crônico de muletas é uma causa rara de aneurisma arterial da região axilobraquial, e alguns casos já foram relatados na literatura^1^^-^ 3. Entretanto, a associação com aneurismas venosos nessa topografia, induzidos pelo uso desse tipo de órtese, tem incidência desconhecida. Não detectamos relatos desse achado na pesquisa da literatura disponível.

Relatamos o caso de uma paciente que fez uso contínuo de muleta por 48 anos, admitida com quadro de isquemia aguda de membro superior esquerdo devido a trombose de aneurisma da artéria braquial proximal e achado incidental de aneurismas da veia braquial.

## DESCRIÇÃO DO CASO

**Figura 1:**
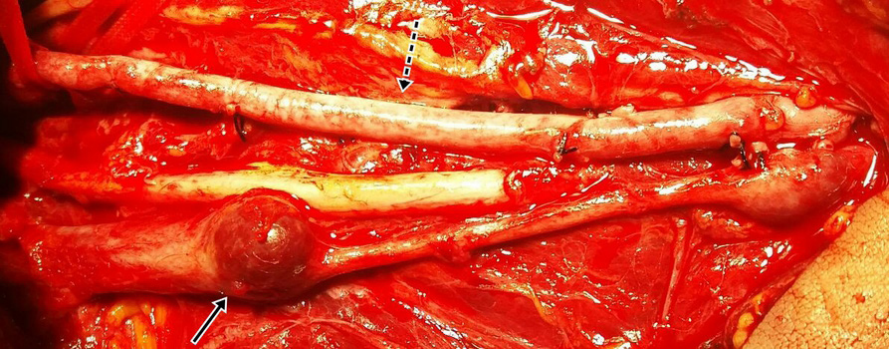
Reconstrução da artéria braquial esquerda com veia safena magna invertida (seta pontilhada) e aneurismas em veia braquial, com destaque para o proximal (seta contínua).

**Figura 2:**
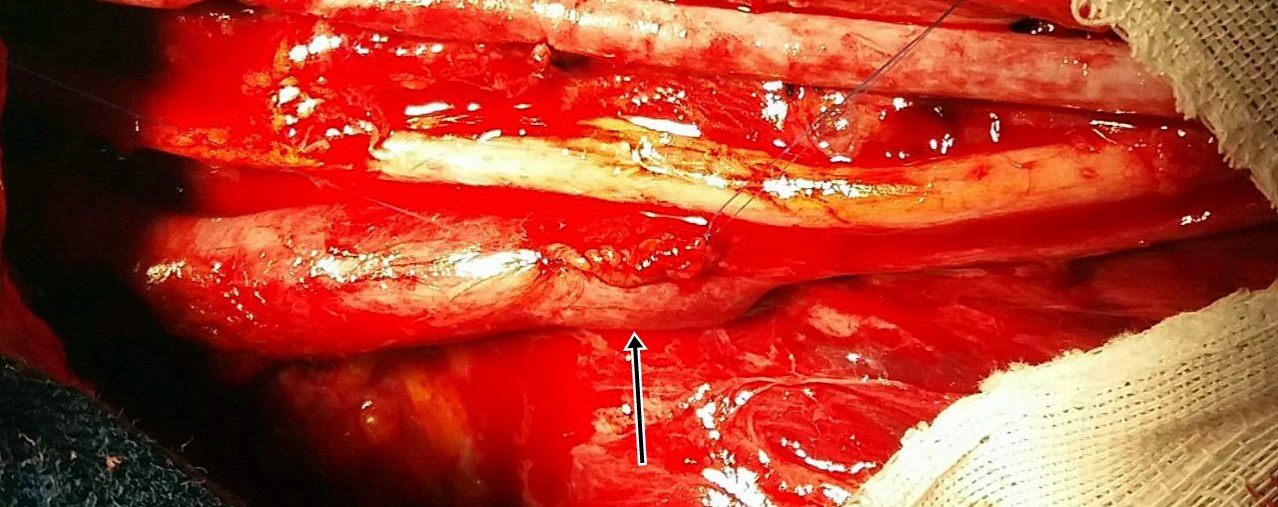
Aspecto final após rafia lateral realizada no aneurisma proximal da veia braquial esquerda.

Paciente do sexo feminino, 68 anos de idade, admitida com quadro de isquemia do membro superior esquerdo evoluindo havia 18 dias, com piora importante ocorrendo no dia anterior à internação, sem relato de sintomas isquêmicos prévios. Ao exame físico, não havia alterações nas auscultas cardíaca e pulmonar. O membro superior esquerdo (MSE) apresentava palidez e frialdade em antebraço, pulso axilar, e ausência dos pulsos braquial, radial e ulnar. Tinha história pregressa de hipertensão arterial sistêmica, fibromialgia e sequela motora no membro inferior esquerdo devido à poliomielite, fazendo uso de muleta por 48 anos, apoiada sobre região axilobraquial esquerda. A ultrassonografia com Doppler e a angiotomografia evidenciaram aneurisma trombosado da artéria braquial esquerda (7 cm de extensão por 3 cm de diâmetro anteroposterior), reenchimento arterial distal por colaterais e identificação de dois aneurismas na veia braquial (um proximal com 2,5 cm de diâmetro anteroposterior e 3 cm de extensão e outro distal com 1,5 cm de diâmetro anteroposterior e 1,5 cm de extensão). Foi encaminhada ao centro cirúrgico e submetida emergencialmente a embolectomia por cateter-balão, com acesso na fossa antecubital, levando à saída de trombos da artéria radial, porém sem progressão do cateter-balão pela artéria ulnar, distalmente. Ao término da cirurgia, identificou-se pulso radial de boa amplitude. No segundo dia pós-operatório, ela foi reencaminhada ao centro cirúrgico para tratamento definitivo. Foi realizada a ressecção do segmento aneurismático arterial e reconstrução terminoterminal com veia safena magna ipsilateral reversa, rafia lateral do aneurisma venoso proximal (Figura 1) e fasciotomia volar do antebraço esquerdo. A aneurismorrafia venosa foi realizada com sutura contínua longitudinal, obtendo-se, ao seu término, redução da luz aneurismática para o diâmetro venoso proximal (Figura 2). A evolução do caso foi satisfatória, com normalização da perfusão distal. Após a alta hospitalar, a paciente abandonou o uso da muleta, passando a deambular com o auxílio de uma bengala. Durante todo o acompanhamento, não houve novos episódios de obstrução arterial ou venosa.

## DISCUSSÃO

O uso crônico de muleta pode induzir à formação de aneurisma da artéria axilar e/ou braquial devido à sobrecarga de peso e ao trauma contuso local. Embora seja de baixa intensidade, esse trauma é recorrente e cumulativo, provocando ruptura e degeneração da túnica íntima e da túnica média^1^.

Os sinais e sintomas variam de acordo com o tamanho e a localização do aneurisma. A apresentação clínica mais comum é a isquemia súbita do membro superior por trombose, com possibilidade de isquemia crônica ou compressão do plexo braquial. Embora a principal causa de isquemia aguda nos membros superiores seja a embolia de origem cardíaca, diante de um paciente com histórico de uso prolongado de muletas, é necessário suspeitar que a etiologia possa estar associada à lesão vascular induzida pelo uso dessa órtese^1^^,^ 3 ^,^ 4.

O aneurisma da artéria braquial está associado mais frequentemente a microembolizações distais recorrentes de trombos luminais que a outras fontes de êmbolos para o membro superior. O prognóstico em longo prazo é menos satisfatório do que o dos pacientes com outras fontes de embolia, uma vez que as lesões recorrentes podem obstruir gradualmente os vasos distais e comprometer os resultados da revascularização^1^. No caso descrito, embora a artéria ulnar já estivesse ocluída, a perfusão do MSE foi adequadamente sustentada pela artéria radial após a revascularização.

Na literatura, a maioria dos pacientes que desenvolve aneurisma associado ao uso de muletas fez uso desse dispositivo por mais de 30 anos^1^, o que é compatível com o caso relatado. O tratamento inicial, diante de um quadro de isquemia aguda dos membros, objetiva a recanalização precoce. Podem ser consideradas como opções terapêuticas a trombólise ou o tratamento cirúrgico por embolectomia^1^^,^ 2. Após a recanalização, o aneurisma deve ser tratado com ressecção ou exclusão e subsequente reconstrução com enxerto^1^^,^ 4. A fasciotomia do antebraço deve ser considerada um tratamento adjuvante^5^. No presente caso, optou-se pela ressecção do segmento aneurismático com reconstrução arterial através do uso de enxerto da veia safena magna ipsilateral reversa.

Com relação aos aneurismas venosos, sabe-se que são uma entidade incomum, sendo mais prevalentes nos membros inferiores e raramente encontrados nos membros superiores. Devem ser reconhecidos por causa das potenciais complicações inerentes a essa patologia, como trombose venosa profunda (TVP), ruptura e compressão de estruturas adjacentes^6^^,^ 7. Os aneurismas venosos descritos na literatura são, em sua maioria, secundários a trauma, fístula arteriovenosa ou doença varicosa^6^^,^ 8. Não são encontrados casos descritos especificamente relacionados ao uso de muleta. Embora, no presente caso, os aneurismas venosos fossem assintomáticos, eles devem ser considerados no diagnóstico diferencial dado o potencial de complicações em pacientes que usam esse tipo de órtese, especialmente naqueles que apresentam TVP ou tromboembolismo pulmonar^7^.

O tratamento convencional para os aneurismas venosos das extremidades inferiores é a ressecção cirúrgica, devido às taxas elevadas de complicações tromboembólicas associadas^6^^,^ 7. No entanto, o tratamento definitivo dos aneurismas venosos das extremidades superiores é bem menos definido. As opções são ressecção e reconstrução com ou sem enxertia, ligadura, plicatura, rafia lateral ou tratamento conservador com anticoagulação em pacientes com risco elevado de tromboembolismo venoso^6^. Ainda não está claro se o manejo cirúrgico agressivo é a melhor forma de tratar, particularmente em pacientes assintomáticos, uma vez que a ressecção não é isenta de complicações^6^^,^ 9. De modo geral, o tratamento definitivo não é uniforme e deve ser individualizado, considerando o risco cirúrgico do paciente e a identificação ou não de sintomas^6^^,^ 9.

A prevenção deve ser enfatizada com o objetivo de eliminar a pressão sobre a região axilobraquial através do estímulo ao uso de dispositivos mais ergométricos e seguros. Desse modo, evita-se a lesão vascular induzida pelo uso de muleta, suas complicações e as sequelas definitivas^1^^,^ 9.
