# Supplementary material for: Aneurismas da artéria e da veia braquial induzidos por uso contínuo de muleta: relato de caso
Source: J Vasc Bras. 2017 Oct-Dec;16(4):325–8. [Article in Portuguese] doi: 10.1590/1677-5449.005317 (PMC5944311; doi:10.1590/1677-5449.005317)
Supplement: Translation

# Translation: Aneurysms of brachial artery and vein induced by continuous use of crutch: case report

**Authors:** Débora Louise Lopes da Costa, Geraldo Felipe, Marcos Aurélio Perciano Borges

**Affiliations:** 1 Hospital de Base do Distrito Federal – HBDF, Unidade de Cirurgia Vascular e Angiologia, Brasília, DF, Brazil.

**Keywords:** vascular system injuries, aneurysm, brachial artery

## Abstract

Crutch-induced arterial aneurysm is a rare event and there are no descriptions in the literature of cases with concomitant venous aneurysms. We report the case of a patient who, after prolonged crutch use, presented with acute ischemia of the upper limb secondary to brachial artery aneurysm thrombosis, associated with the incidental finding of brachial vein aneurysms. Although the main cause of acute upper limb occlusion is embolization of cardiac origin, consideration should be given to the possibility of arterio-arterial embolization due to an aneurysm induced by prolonged use of crutches. Venous aneurysms should also be suspected since they can be sites of thrombosis, and a source of pulmonary embolism.

## INTRODUCTION

Chronic crutch use is a rare cause of arterial aneurysm in the axillobrachial area and some cases have been described in the literature previously.^1^^-^ 3 However, the incidence of arterial aneurysm combined with venous aneurysms in the same topography and induced by crutch use is unknown. We were unable to identify reports of this combination of findings in searches of the available literature.

Here we describe the case of a patient who had continuously used a crutch for 48 years and was admitted to hospital with acute ischemia of the left upper limb caused by thrombosis of an aneurysm of the proximal brachial artery, in whom aneurysms were also detected incidentally in the brachial vein.

## CASE DESCRIPTION

A 68-year-old female patient was admitted for investigation of ischemia of the left upper limb with onset 18 days previously, significant exacerbation the day prior to admission, and no previous history of ischemic symptoms. During physical examination, cardiac and pulmonary auscultation were unremarkable. The forearm of the left upper limb was pale and cool, the axillary pulse was present, but brachial, radial and ulnar pulses were absent. The patient had a previous history of systemic arterial hypertension, fibromyalgia, and sequelae of poliomyelitis causing impaired motor function in the left lower limb, for which she had been using a crutch for 48 years, which she supported against her left axillobrachial area. Doppler ultrasonography and angiotomography revealed evidence of a thrombosed aneurysm of the left brachial artery (7 cm in length by 3 cm in anteroposterior diameter), filling of distal arteries by collateral circulation and two aneurysms in the brachial vein (one more proximal with an anteroposterior diameter of 2.5 cm and a length of 3 cm and the other more distal with anteroposterior diameter of 1.5 cm and length of 1.5 cm). The patient was transported to the operating theatre and underwent emergency embolectomy with a balloon catheter, via an antecubital fossa access, which caused the thrombi to exit the radial artery, but with no progression of the balloon catheter distally through the ulnar artery. At the end of surgery, the radial pulse was present and strong. On the second day after the operation, the patient was once more taken to the operating theater for definitive treatment. The aneurysmal segment of the artery was resected and reconstructed with an end-to-end graft using the ipsilateral great saphenous vein reversed, followed by lateral venorrhaphy of the proximal venous aneurysm (Figure 1) and volar fasciotomy of the left forearm. Venous aneurysmorrhaphy was accomplished with continuous longitudinal sutures and at the end the aneurysm lumen had been reduced to the diameter of the proximal vein (Figure 2). The patient’s recovery was satisfactory, with normalization of distal perfusion. After discharge from hospital, the patient stopped using the crutch and began using a walking stick to help her walk. There were no further episodes of arterial or venous obstruction at any point during follow-up.

**Figure 1:** Reconstruction of the left brachial artery with the great saphenous vein reversed (dotted arrow) and aneurysms in the brachial vein, of which the proximal is more notable (arrow continuous).

**Figure 2:** Final appearance after lateral venorrhaphy of the proximal aneurysm of the left brachial vein.

## DISCUSSION

Chronic use of a crutch can induce formation of aneurysms of the axillary and/or brachial arteries in response to the weight overload and local blunt trauma. While the trauma is of low intensity, it is recurrent and cumulative, provoking rupture and degeneration of the tunica intima and the tunica media.^1^

Signs and symptoms vary depending on the size and site of the aneurysm. The most common clinical presentation is sudden ischemia of the upper limb caused by thrombosis, and chronic ischemia or compression of the brachial plexus are also possible. Although the principal cause of acute ischemia of the upper limbs is embolism of cardiac origin, when a patient has a history of prolonged crutch use, it is necessary to suspect that etiology could be associated with vascular injury caused by crutch use.^1^^,^ 3 ^,^ 4

Brachial artery aneurysms are more often associated with recurrent distal microembolizations of luminal thrombi than with other sources of emboli in the upper limb. Prognosis over the long term is less satisfactory than for patients with other sources of embolism, since recurrent lesions can gradually obstruct the distal vessels and compromise the results of revascularization.^1^ In the case described here, although the ulnar artery was already occluded, adequate perfusion of the limb was maintained by the radial artery after revascularization.

The majority of patients in the literature who developed aneurysms related to use of crutches had been using the device for more than 30 years,^1^ which is compatible with the case reported. The initial treatment in cases with acute limb ischemia is focused on early recanalization. Treatment options that can be considered include thrombolysis or surgical treatment with embolectomy.^1^^,^ 2 After recanalization, the aneurysm should be treated by resection or exclusion, followed by reconstruction with grafting.^1^^,^ 4 Fasciotomy of the forearm should be considered as an adjuvant treatment.^5^ In the present case, it was decided to conduct resection of the aneurysmal segment and arterial reconstruction with the ipsilateral great saphenous vein reversed.

With regard to the venous aneurysms, it is known that they are an uncommon entity that are most prevalent in the lower limbs and are rarely found in the upper limbs. They should be recognized as causes of the potential complications inherent to this pathology, such as deep venous thrombosis (DVT) and rupture and compression of adjacent structures.^6^^,^ 7 The majority of venous aneurysms described in the literature are secondary to trauma, arteriovenous fistula or varicose disease.^6^^,^ 8 No cases were found that specifically describe a relation to crutch use. Even though the venous aneurysms were asymptomatic in this case, they should be taken into consideration during differential diagnosis, given the potential for complications in patients who use crutches, especially those who develop DVT or pulmonary thromboembolism.^7^

The conventional treatment for venous aneurysms in the lower limbs is surgical resection, because of the high rates of thromboembolic complications associated with them.^6^^,^ 7 However, the definitive treatment for venous aneurysms in the upper extremities is much less well defined. The options are resection and reconstruction with or without grafting, ligature, plication, lateral venorrhaphy, or conservative treatment with anticoagulation in patients at high risk of venous thromboembolism.^6^ It is not yet clear whether aggressive surgical treatment is the best option, particularly with regard to asymptomatic patients, since resection is not free from complications.^6^^,^ 9 In general, the definitive treatment is not uniform and should be chosen on a case-by-case basis considering the patient’s level of surgical risk and whether or not symptoms are identified.^6^^,^ 9

Prevention should be prioritized, with the objective of eliminating pressure on the axillobrachial area by encouraging the patient to use devices that are more ergonomic and safer. This can avert the crutch-induced vascular injury, its complications and its definitive sequelae.^1^^,^ 9
